# Incidence and long-term outcomes of dextro-transposition of the great arteries (d-TGA) in the Kingdom of Bahrain

**DOI:** 10.3389/fcvm.2025.1691860

**Published:** 2025-11-17

**Authors:** Ahmed J. AlAraibi, Fatema Naser Shakeeb, Zahra Naser Shakeeb, Salim Fredericks, Aditya Bhat, N. N. Kalis

**Affiliations:** 1 Royal College of Surgeons in Ireland, Medical University of Bahrain (RSCI-MUB), Busaiteen, Bahrain; 2 Mohammed bin Khalifa bin Salman Al-Khalifa Cardiac Center, Pediatric Cardiology, Awali, Bahrain

**Keywords:** dextro-transposition of the great arteries, congenital heart disease, arterial switch operation, Rastelli, consanguinity, IDM

## Abstract

**Background:**

Dextro-Transposition of the Great Arteries (d-TGA) is a rare cyanotic congenital heart defect requiring surgical correction for survival. The arterial switch operation (ASO) is the preferred approach, offering favorable long-term outcomes. However, long-term outcomes from the Gulf Cooperation Council (GCC) region remain largely unreported. This study presents the first national cohort from GCC, evaluating long-term surgical outcomes of d-TGA.

**Methods:**

A retrospective cohort study was conducted from 1983 to 2025. Patients were classified based on associated anomalies: simple d-TGA without major associated cardiac anomalies, d-TGA with ventricular septal defect (VSD), and d-TGA with left ventricular outflow tract obstruction (LVOTO). Kaplan–Meier analysis assessed survival and event-free survival (EFS).

**Results:**

88 patients were included (male-to-female ratio 1.6:1). 20 were born to diabetic mothers, and 19 to consanguineous parents. Incidence rose over time, peaking at 2.58 per 10,000 live births. Median age at surgery declined to 27 days. Early mortality was 4.5%, with one postoperative ASO death. Reintervention was required in 35.2% of cases. Median EFS was highest in simple d-TGA (20.6 years), lowest after Rastelli repair (0.5 years). d-TGA with obstruction was statistically significant associated with postoperative left ventricular outflow obstruction (LVOTO), and simple d-TGA with right ventricular outflow obstruction (RVOTO).

**Conclusion:**

This is the first GCC-based study reporting long-term d-TGA outcomes. ASO remains the most effective intervention with superior survival and EFS. Consanguinity and maternal diabetes were key risk factors. Early diagnosis, close follow-up, and targeted public health interventions are essential to improving outcomes.

## Introduction

Dextro-Transposition of the Great Arteries (d-TGA) is a rare cyanotic congenital heart defect requiring surgical correction. It involves atrioventricular concordance with ventriculoarterial discordance: the right atrium connects to the right ventricle, which gives rise to the aorta, while the left atrium connects to the left ventricle, which gives rise to the pulmonary artery (1). Representing 2% of all cyanotic congenital heart diseases (2), the incidence of d-TGA is 20–30 per 100,000 live births and a male predominance (male-to-female ratio 1.5:1–3.2:1) (3, 4, 5).

In 1948, the first palliative operation, atrial septostomy, was introduced by Hanlon and Blalock. In 1953, Lillhei and Marco performed the partial venous switch. The atrial switch procedure was further developed in 1955 by Senning, who used atrial flaps, followed by Mustard in 1963, who employed pericardium for the procedure. Finally, in 1975, the successful arterial switch operation was pioneered by Jatene et al. This breakthrough was transformative in the history of d-TGA, as it marked a major shift towards corrective surgery, significantly improving the prognosis and long-term outcomes for patients (6). Its superiority is supported by long-term outcomes demonstrating the preservation of good left ventricular function, sinus rhythm, and low mortality rates (7, 8).

In the ASO era, in-hospital mortality is exceptionally low, with a 20-year survival rate approaching >97% (9). Long-term outcomes are excellent, with 93% survival at 35 years. This contrasts with a 64% survival at 40 years reported in a meta-analysis of d-TGA patients treated with atrial switch correction (10, 11).

There is a noticeable gap in the literature regarding the long-term survival of d-TGA, in our region. This study aims to explore the long-term outcomes of d-TGA patients within our population.

## Methods

This retrospective cohort study included all patients diagnosed with dextro-Transposition of the Great Arteries (d-TGA) between 1983 and 2025. Data were collected from institutional databases and individual medical records. Patients who underwent surgical correction abroad but received follow-up care in Bahrain were also included and were identified through national registry numbers and cardiology clinic records. Loss to follow-up was defined as absence of clinical records for more than two consecutive years or from the beginning of data collection.

Incidence rates were calculated per 10,000 live births using national birth registry data. For clarity, the cohort was divided into three temporal groups: 2000–2010, 2011–2020, and 2021–2024. These groupings were chosen to reflect decade-based reporting, align with available registry data, and highlight recent trends in diagnosis and management. Live birth numbers for each interval were used as denominators.

Patients who underwent definitive surgical correction were categorized into three diagnostic groups: simple d-TGA (associated with ASD, PFO, or PDA), d-TGA with ventricular septal defect (VSD) without significant outflow tract obstruction, and d-TGA with left ventricular outflow tract obstruction (LVOTO), with or without associated VSD. Long-term survival and event-free survival (EFS), defined as time to death or reintervention, were analyzed across these diagnostic subgroups and surgical procedures.

Surgical repairs were performed in line with accepted international norms. Patients with simple d-TGA who presented early were managed with an arterial switch operation (ASO), including coronary artery transfer, the LeCompte maneuver, and closure of associated shunts such as VSDs or PDAs when required. Complex d-TGA with left ventricular outflow tract obstruction (LVOTO) was treated with the Rastelli procedure, while patients with significant systemic outflow hypoplasia underwent a Damus–Kaye–Stansel (DKS) anastomosis. Atrial switch procedures (Senning/Mustard) were reserved for late presenters with regressed left ventricles, where ASO was no longer feasible. In selected high-risk neonates.

Preoperative management included prostaglandin to maintain ductal patency and balloon atrial septostomy (BAS) for restrictive atrial communication. In selected cases, palliative procedures such as Blalock–Taussig shunts (BTS) or pulmonary artery banding (PAB) were used to improve oxygenation or retrain the left ventricle. Atrial septectomy was used to improve mixing in specific scenarios.

All analyses were performed using SPSS version 29.0. Normality of continuous variables was assessed using the Shapiro–Wilk test. Results indicated non-normal distribution across all primary continuous variables, justifying the use of non-parametric methods. Continuous variables are expressed as median (interquartile range). Two-group comparisons were performed using the Mann–Whitney *U* test, while comparisons across more than two groups were performed with the Kruskal–Wallis test followed by Bonferroni-corrected *post-hoc* testing. Categorical variables are expressed as counts (percentages). The Chi-square test was applied when all expected cell counts were ≥5; otherwise, Fisher's exact test was used. Time-to-event data were analyzed using Kaplan–Meier survival curves, with between-group differences assessed using the log-rank test. A two-sided *p*-value < 0.05 was considered statistically significant.

## Results

### Baseline characteristics

A total of 88 patients with d-TGA met the inclusion criteria between 1983 and 2025. Most were Bahraini (55%), followed by Arab non-Bahraini (31%) and South Asians (13%). The cohort had a male-to-female ratio of 1.6:1. The most common associated anomaly was d-TGA with VSD. 20 patients were born to diabetic mothers, and 19 to consanguineous parents. Additional characteristics are presented in Table 1.

**Table 1:** Baseline demographic and clinical characteristics of participants (*N* = 123).

Variable	*n* (%)	Median (IQR)
Sex		
Male	54 (61.4)	–
Female	34 (38.6)	
Age at diagnosis	–	34 (4–439) days
Nationality		
Bahraini	55 (62.5)	–
Arab Non-Bahraini	17 (19.4)	
Southeast Asians	13 (14.8)	
Others	2 (2.3)	
Type of d-TGA		
Simple d-TGA	24 (27.3)	–
d-TGA with VSD	18 (20.5)	
d-TGA with Obstruction	43 (48.9)	
d-TGA with complications	3 (3.3)	
Associated cardiac anomalies		
VSD	44 (50.0)	—
Supra PS	39 (44.3)	
ASD	40 (45.5)	
COARCT	10 (11.4)	
PDA	32 (36.4)	
PFO	8 (9.1)	
LVOTO	12 (13.6)	
Coronary Circulation anomaly	4 (4.5)	
DEXTROCARDIA	2 (2.3)	
ARCH HYPOPLASIA	6 (6.8)	
IAA	2 (2.3)	
History		
IDM	20 (22.7)	—
CONSANGUINEOUS PARENTS	19 (21.6)	
Age at surgery		27 (16–77.25) days
Lost to follow-up post OP	11 (12.5)	–
Expired before surgical correction	3 (3.4)	–
Curative repair	51 (58.0)	–
Palliative repair (PA band + Atrial septectomy or BTS)	14 (15.9)	–
Staged repair	2 (2.3)	–
BAS before repair	6 (6.8)	–
Meds from initial visit		
PGE	29 (33.0)	–
Furosemide	4 (5)	
Amiodarone	2 (2.3)	
Adenosine	2 (2.3)	
Digoxin	1 (1.1)	
Aspirin	1 (1.1)	
Sildenafil	1 (1.1)	
Atenolol	1 (1.1)	

ASD, atrial septal defect; PDA, patent ductus arteriosus; PFO, patent foramen ovale; COARCT, coarctation of the aorta; LVOTO, left ventricular outflow tract obstruction; IAA, interrupted aortic arch; IDM, infant of diabetic mother; BAS, balloon atrial septostomy; BTS, Blalock–Taussig shunt; PGE, prostaglandin E.

### Incidence

The incidence of d-TGA increased over the study period, from 1.1 per 10,000 live births (2000–2010) to 1.5 (2011–2020), peaking at 2.58 per 10,000 in 2021–2024.

### Age at surgery

**Figure 1:**
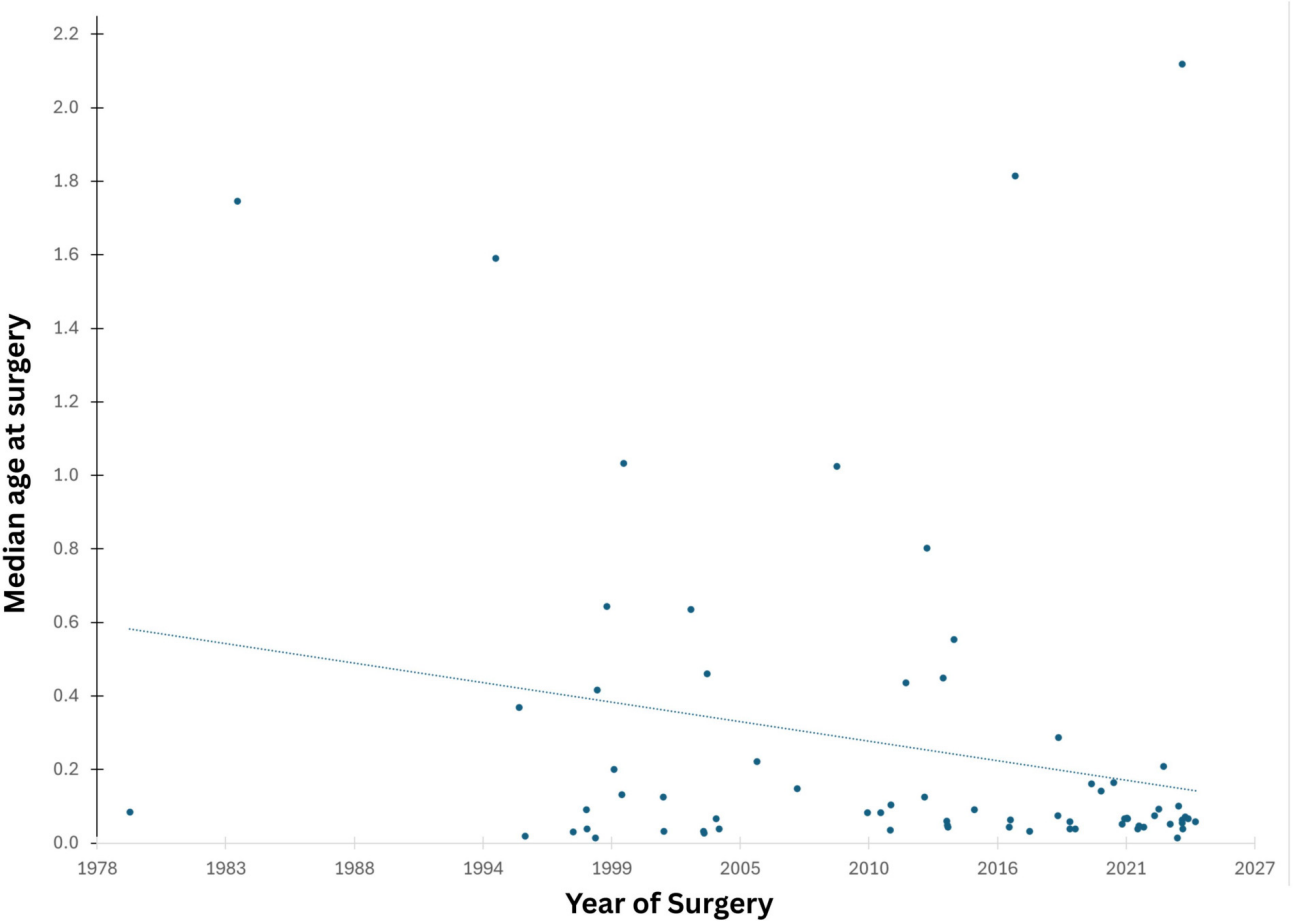
Trend of Age of first surgery over years.

Age at surgery declined significantly over time from ∼6 months in the early 2000s to ∼1 month in recent years. The current median age at surgery is 27 days (Figure 1).

### Types of surgery

A total of 69 patients underwent surgical intervention for d-TGA, including ASO, atrial switch, Rastelli, BTS, and PA banding with atrial septostomy. ASO was the most common (*n* = 48), with anatomical subtypes: simple d-TGA (25%), d-TGA with VSD (25%), and d-TGA with outflow tract obstruction (50%). Reintervention was required in 35% of ASO patients, with a mean EFS of 22.5 ± 3.3 years.

Atrial switch (*n* = 6) was mostly used for simple d-TGA (84%), with a high reintervention rate (83%) and lower mean EFS of 15 ± 3.1 years. The Rastelli procedure (*n* = 3), used exclusively for d-TGA with outflow tract obstruction, had a 100% reintervention rate and the shortest mean EFS (1.9 ± 1.4 years).

Palliative procedures including BTS (*n* = 7) and PA banding with atrial septostomy (*n* = 4) were used in complex cases, with reintervention rates of 43% and 75%, and mean EFS of 11.4 ± 4.6 and 14.3 ± 2.6 years, respectively.

Overall, ASO yielded the best long-term outcomes.

### Mortality

**Figure 2:**
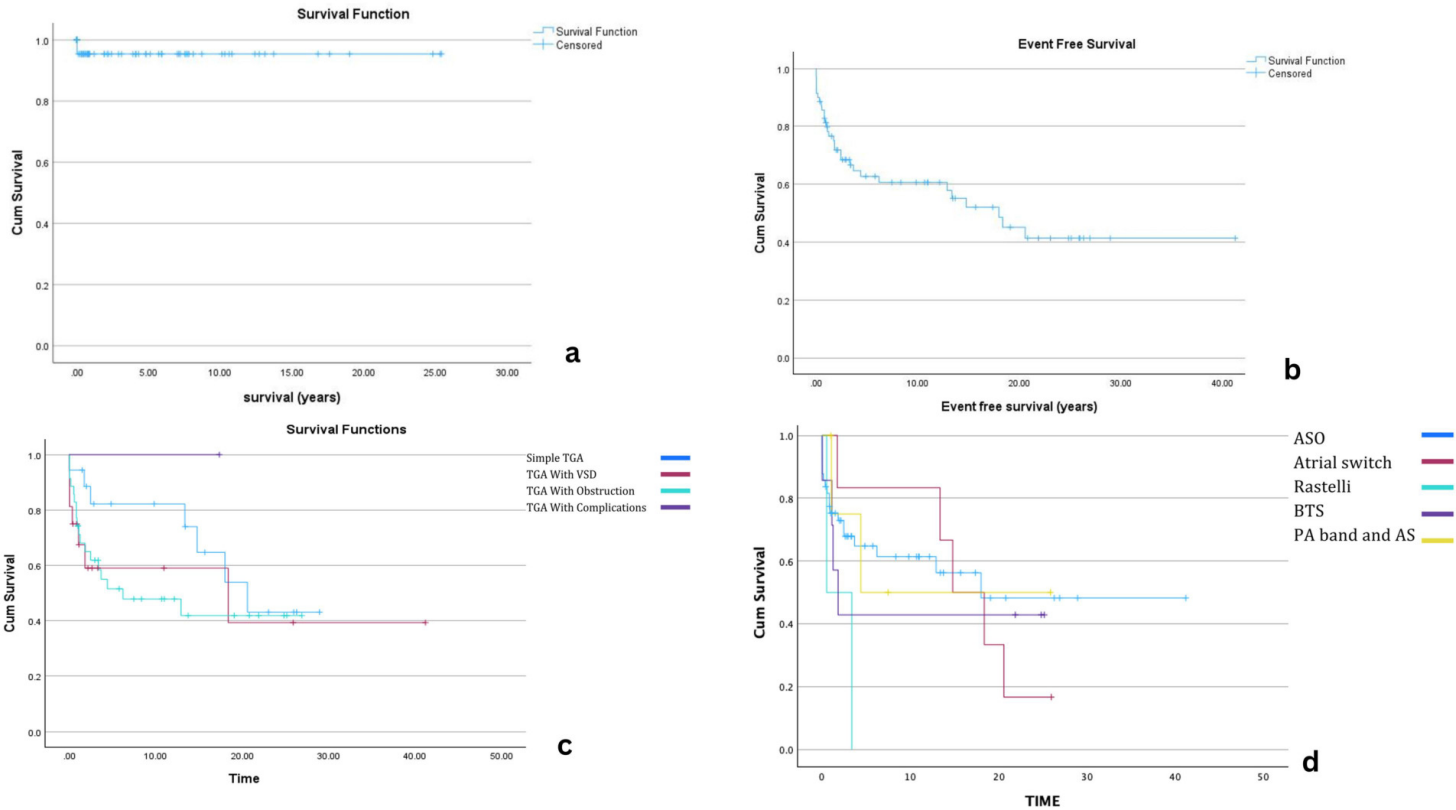
Kaplan–Meier survival analyses for patients with dextro-transposition of the great arteries (d-TGA). **Panel (a)** Overall survival following diagnosis. **Panel (b)** Event-free survival (EFS) for all patients after surgical repair. **Panel (c)** EFS stratified by surgical procedure (arterial switch operation, atrial switch, Rastelli). **Panel (d)** EFS stratified by diagnostic subtype (simple d-TGA, d-TGA with VSD, d-TGA with LVOTO). Censored observations are indicated by “+”.

Overall survival after diagnosis with d-TGA is showed on KM graph. Figure 2. Early mortality was documented in four patients (4.5%). Three patients expired prior to surgical correction. One neonate, born prematurely with a birth weight of 1.7 kg, with associated mitral regurgitation (MR) died on the first day of life with suspected pulmonary hypertension (PHT); however, no definitive cause of death was identified. A second patient, who had a single coronary artery, remained clinically stable on prostaglandin E2 (PGE2) for three days but experienced sudden cardiac arrest, with a presumed arrhythmic event. The third patient, an infant, developed persistent pulmonary hypertension (PPHT) following a failed balloon atrial septostomy (BAS) and succumbed despite maximal supportive care.

One patient died postoperatively following ASO. No specific details on the cause of death were available, but this patient had associated aortic arch hypoplasia.

### Event-free survival

EFS varied based on the type of d-TGA diagnosis. The overall median EFS for patients undergoing surgical intervention was 14.8 years (95% CI: 8.689–20.875). Patients with simple d-TGA had the most favorable outcomes, with a median EFS of 20.6 years, followed by those with d-TGA and VSD (18.4 years). In contrast, patients with d-TGA and outflow tract obstruction had significantly lower EFS (6.2 years), indicating a higher likelihood of reintervention.

**Figure 3:**
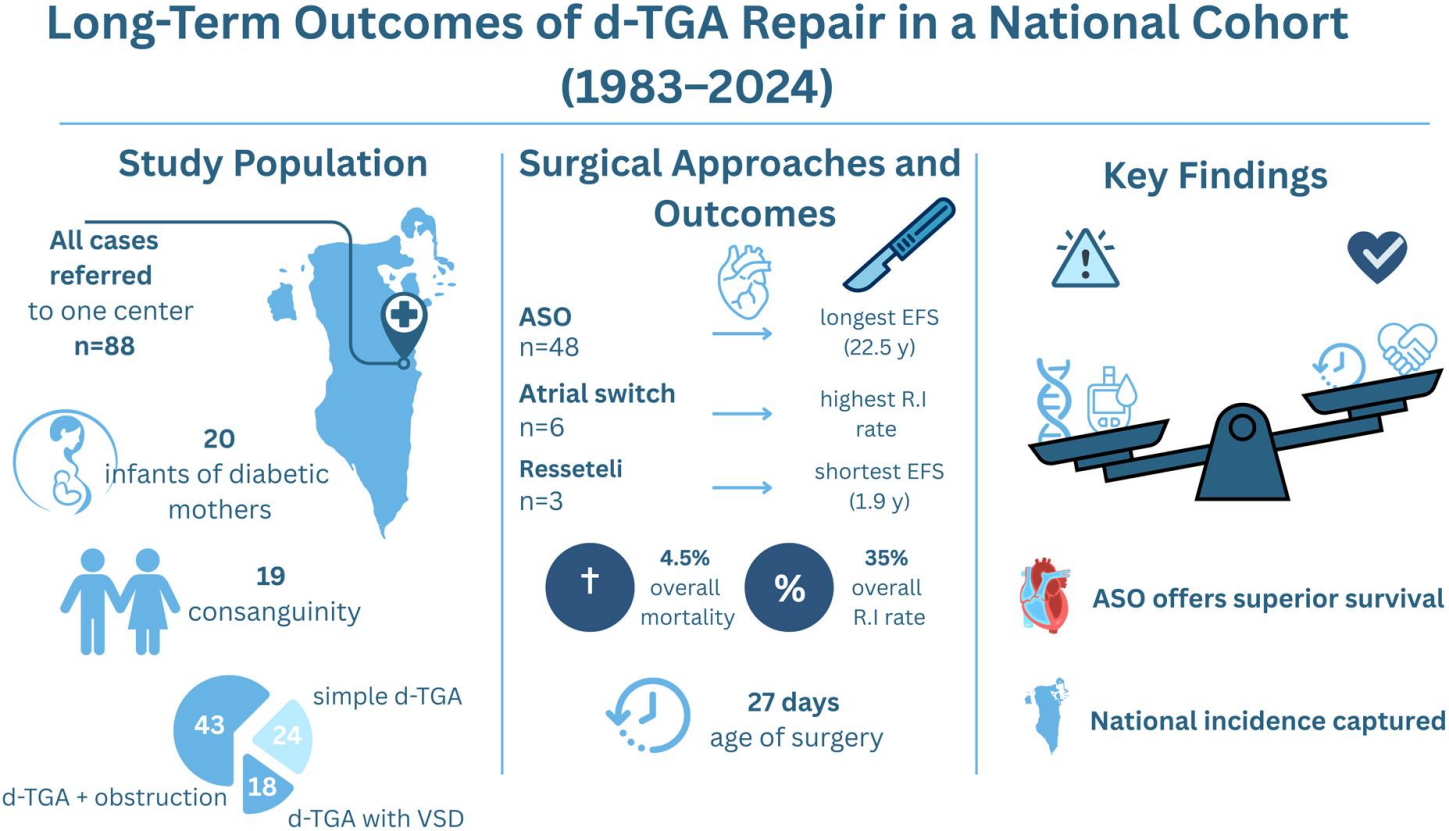
Central illustration—long-term outcomes of d-TGA repair in a national cohort (1983–2024). Left panel: Cohort demographics, including 88 patients referred to a single center, 20 infants of diabetic mothers, and 19 from consanguineous families. Middle panel: Event-free survival by procedure, with arterial switch operation (ASO) showing the longest durability (22.5 years) compared with atrial switch and Rastelli procedures. Overall mortality was 4.5%, and the reintervention rate was 35%. The median age at surgery declined to 27 days in recent decades. Right panel: Balance of risk and protective factors influencing outcomes, emphasizing ASO as the superior surgical strategy.

Among procedures, ASO yielded the most favorable long-term outcomes with a median EFS of 18 years. In comparison, the Rastelli procedure had the shortest EFS (0.5 years). Atrial switch patients (Senning/Mustard) had a median EFS of 14.8 years; BTS patients had a median EFS of 1.8 years, and those who underwent PA banding with atrial septectomy had an EFS of 4.4 years. These findings highlight the variability in outcomes depending on the initial surgical approach, with ASO demonstrating the most durable results. Detailed comparisons are shown in Kaplan–Meier graphs (Figure 3) and numbers at risk (Tables 2, 3).

**Table 2:** Number at risk by surgery type.

Time (years)	ASO	Atrial switch	Rastelli	BTS	PA
0	48	6	2	7	4
5	19	6	1	5	3
10	13	3	0	4	2
15	8	2	0	3	1
20	4	1	0	1	0

**Table 3:** Number at risk by d-TGA subtype.

Time (years)	Simple d-TGA	d-TGA with VSD	d-TGA with obstruction	d-TGA with complications
0	20	17	42	1
5	14	13	38	1
10	11	8	18	1
15	9	6	16	1
20	7	3	12	1

During follow-up, nine patients developed right ventricular outflow tract obstruction (RVOTO), with one requiring percutaneous pulmonary valve implantation (PPVI). Five developed LVOTO, and three developed pulmonary hypertension. Heart failure due to LV dysfunction was observed in seven patients, all managed medically. Additionally, three patients had peripheral pulmonary stenosis, and two developed valvular disease, including aortic and mitral regurgitation.

A significant association was observed between d-TGA type and RVOTO development [*χ*^2^(3) = 10.401, *p* = 0.015; Fisher's Exact Test: *p* = 0.015]. Patients with simple d-TGA had a higher-than-expected RVOTO rate (29.4%), while those with d-TGA and obstruction had a lower-than-expected rate (5.7%), indicating that anatomical subtype significantly influences RVOTO development. These findings align with the expected impact of the Lecompte maneuver in ASO patients.

A statistically significant association also emerged between preoperative LV obstruction and postoperative LVOTO [*χ*^2^(1) = 6.205, *p* = 0.013; Fisher's Exact Test: *p* = 0.025]. Patients with preoperative obstruction had a significantly higher postoperative LVOTO rate, with a 1.207-fold increased risk (95% CI: 1.038–1.403).

### Reinterventions

Reoperations were performed in 31 of 69 surgical patients, with six requiring a second reoperation. No early or late mortality was observed among those undergoing a single reoperation. Over half (55%, 17 patients) occurred within the first year, and seven occurred within 30 days. Reoperations were significantly less frequent in patients with simple d-TGA.

Among the 31 reoperated patients, six had simple d-TGA, five had d-TGA with VSD, and 17 had d-TGA with obstruction. RVOT pathology was a major cause, with pulmonary artery and/or valve procedures performed in seven patients. PA banding with atrial septectomy was performed in four, and five required LVOTO resection. PA plasty was done in three patients across all subtypes. VSD closure and definitive repair were performed in two patients with d-TGA and VSD.

Additional procedures included PDA ligation (1 patient), BTS (2 patients), and repair of recoarctation (ReCoA) in one patient. Permanent pacemaker (PPM) implantation was required in three patients (one with simple d-TGA and two with d-TGA and VSD). Aortic valve replacement (AVR) was performed in one patient with simple d-TGA.

Among ASO patients, 17 of 48 required reintervention. Of the six atrial switch patients, five required reinterventions. All three Rastelli patients underwent redo surgery. Three of seven BTS patients also required further surgical procedures. Surgical details by procedure and reintervention summaries are shown in Tables 4, 5.

**Table 4:** Surgical details of d-TGA patients categorized according to the type of surgery.

Procedure (n)	Simple d-TGA	d-TGA with VSD	d-TGA + obstruction	Total reintervention	HF	RVOTO	PPS	Mean EFS time 19.939 ± 2.571	Mean age at surgery	Mean FU
ASO (48)	12 (25%)	12 (25%)	24 (50%)	17 (35%)^a^	3 (6%)	8 (17%)	3 (6%)	22.5 ± 3.3	91.6 ± 156.9	7.0 ± 8.4
Atrial switch operation (6)	5 (84%)	1 (16%)	0 (0%)	5 (83%)^b^	1 (17%)	0 (0%)	0 (0%)	15 ± 3.1	76.9 ± 156.2	25.1 ± 5.4
Rastelli (3)	0 (0%)	0 (0%)	3 (100%)	3 (100%)^b^	1 (33%)	1 (33%)	0 (0%)	1.9 ± 1.4	—	7.3 ± 7.2
BTS (7)	0 (0%)	1 (14%)	6 (86%)	3 (43%)^b^	2 (29%)	0 (0%)	0 (0%)	11.4 ± 4.6	—	7.9 ± 8.6
PA banding + atrial septostomy (4)	0 (0%)	2 (50%)	2 (50%)	3 (75%)^b^	0 (0%)	0 (0%)	0 (0%)	14.3 ± 2.6	—	7.0 ± 10.5

a *p* < 0.05 vs. non-ASO procedures.

b *p* < 0.05 vs. ASO (Chi-square/Fisher's exact test).

**Table 5:** Total number of all procedures performed during reoperation in each patient group.

Procedure	Simple d-TGA (total 6)	d-TGA with VSD (total 5)	d-TGA with obstruction (total 17)
PS and PV	1		6
PA bandging and atrial spetcotmy	2		2
PDA ligation			1
PA plasty	1	1	1
LVOTO resection			5
VSD closure			2
Definitive repair		2	
PPM	1	2	
RECOA			1
BTS			2
AVR	1		

A significant association was found between surgery type and postoperative events [*χ*^2^(1) = 4.997, *p* = 0.025; Fisher's Exact Test: *p* = 0.036]. Non-ASO procedures had a significantly higher event rate (66.7%) compared to ASO (37.5%). The event rate in ASO patients was lower than expected.

### Follow-up

Complete postoperative follow-up was achieved in 84% of patients, with follow-up durations ranging from 1 to 25 years (median: 4.3 years). Eleven patients were lost to follow-up, mainly due to relocation or seeking care abroad. Patients were monitored via echocardiography and ECG, with cardiac catheterization performed selectively for further assessment or intervention.

## Discussion

A male-to-female ratio of 1.6:1 was observed in our study, consistent with existing literature that reports a male predominance in d-TGA. For instance, a large exome-based case-control study by Blue et al. (2020) found a male-to-female ratio of 2.85:1 in d-TGA cases, reinforcing the trend of male overrepresentation in this condition (12). Ventricular Septal Defect (VSD) was the most frequent associated cardiac anomaly in our cohort, consistent with previous studies (13). Long-term outcome comparisons of d-TGA in Bahrain are limited, as follow-up data from Middle Eastern countries remain scarce. Consanguinity was present in 19 patients, reflecting its prevalence in the Middle East. Unlike many studies centered on Western populations, our findings highlight consanguinity as a significant risk factor for transposition of the great arteries (d-TGA), underscoring the need for focused genetic research in this region (14). 20 patients were infants of diabetic mothers (IDM). While maternal diabetes is a known teratogen increasing congenital heart defect risks, including d-TGA and VSD, our findings reinforce this link. The elevated IDM proportion highlights the critical need for strict maternal glycemic control and early prenatal monitoring to minimize congenital cardiac anomalies (15, 16).

Our study found an incidence rate of 1.5 per 10,000 live births for d-TGA, which is slightly lower than reported estimates of 1 in 3,500–5,000 (approximately 2–2.8 per 10,000). Such variation may reflect regional differences, reporting practices, or diagnostic capabilities (1).

We observed a shift toward earlier surgical intervention over time, with the median age at surgery declining from around 6 months in the early 2000s to 27 days in recent years. This improvement aligns with the development of a dedicated paediatric cardiology program and fetal services that have enabled earlier diagnosis and management, consistent with findings in similar studies (17, 18).

Since 1998, d-TGA surgery has shifted earlier, now with a median age of 27 days. Early surgery improves outcomes, while delays increase hospital stay, costs, and healthcare burden (19). Even neonates with normal oxygen levels and a patent ductus arteriosus may have cerebral oxygen below 50%, risking white matter injury from hypoxemia (20, 21). Recent research supports PGE therapy to keep the ductus open, echocardiographic monitoring, and NIRS to assess cerebral oxygen saturation non-invasively (22).

Our early mortality rate was 4.5%, similar to a nationwide rate of 3.3% (23, 24). Unlike studies showing no survival difference (9, 25), we found a significant survival benefit with ASO. Despite common reinterventions, ASO provides true anatomical correction and better long-term outcomes. Simple ASO had the highest 18-year event-free survival, supporting ASO as the gold standard for d-TGA with superior survival and long-term results (25, 26).

Although the atrial switch has a high survival rate, our study showed a 35% reintervention rate (11). ASO remains a safe and reliable procedure for d-TGA, with a low 30-day mortality rate of 0.9% (27). Despite its safety, right ventricular outflow tract obstruction, a leading cause of reintervention, was a significant postoperative challenge in our cohort (28). Left ventricular outflow tract obstruction was also observed as a reintervention cause. Notably, no deaths occurred post-reintervention, highlighting the overall effectiveness and safety of ASO and aligning with other studies that reinforce its long-term reliability (29).

Similar studies show that pacemaker implantation after atrial switch procedures particularly, Senning and Mustard, is linked to poorer outcomes. In our study, 2 of 3 patients needing pacemakers had undergone the Senning procedure. Reoperation risk is influenced not only by the procedure but also by surgical experience and pacemaker need. In our cohort, 5 of 6 patients (83%) required reoperation, higher than reported elsewhere. This may reflect limited expertise or infrequent use of the atrial switch technique (30).

The Rastelli procedure is associated with higher reoperation risk and residual pulmonary outflow tract obstruction. Studies report increased early postoperative mortality, frequent reoperations, and a higher incidence of arrhythmias (31). In our study, all three patients who underwent Rastelli required reintervention, primarily due to homograft replacement. This is largely because the conduit does not accommodate the patient's somatic growth, leading to obstruction and the need for surgical revision.

In our study, 3 of 7 patients who underwent the Blalock-Taussig (BT) shunt required reintervention. Reintervention is influenced by left ventricular mass and pressure post-shunt. If heart failure is absent or manageable, later anatomical correction may be possible. However, in cases of uncontrolled heart failure, the shunt should be removed, and arterial or venous switch performed promptly (32).

Simple d-TGA had the lowest reintervention rate at 25%, followed by d-TGA with VSD at 27%, and d-TGA with obstruction had the highest at 39%. These results align with previous studies on survival and reintervention rates in d-TGA (33, 34, 35). Our study found that right ventricular outflow tract obstruction (RVOTO) is the most common postoperative complication in simple d-TGA, whereas left ventricular outflow tract obstruction (LVOTO) occurs more frequently in d-TGA with obstruction. These observations support previous evidence (1, 2, 36).

The median follow-up duration was 4.3 years, consistent with other studies (34). A significant portion of patients are non-Bahraini and return to their home countries, complicating long-term follow-up. Historical resource limitations necessitated overseas referrals, further impacting follow-up. The establishment of a tertiary cardiac center has alleviated this issue. Despite these challenges, event-free survival aligns with existing literature, with simple d-TGA showing the highest rates (29).

In our cohort, 17 of 48 patients (35%) who underwent arterial switch operation (ASO) required reintervention, with a mean event-free survival of 22.5 ± 3.3 years, while overall survival after ASO was excellent at 97.9%. Compared to the Austrian SUTRA cohort, which reported 10-year survival rates of 97.9% in the most recent era (2006–2020), our survival outcomes are comparable, although our reintervention rate is higher than typically reported in contemporary European series. These findings suggest that, despite a higher frequency of reinterventions, timely management can preserve excellent long-term survival, aligning our results with outcomes from large European registries (37).

The study retrospective design and extended period (42 years) introduce variability due to evolving diagnostic tools, surgical techniques, and criteria for reintervention that enables long-term outcome evaluation in a rare congenital condition and reflects evolving surgical practices over time. However, this wide temporal range also introduces variability in care, which represents a key limitation. Loss to follow-up in a subset of patients may have influenced survival and reintervention estimates. Additionally, because many patients underwent surgery in more recent years, long-term follow-up beyond two decades was limited for a portion of the cohort, which may impact interpretation of late outcomes.

Despite the 42-year span of the cohort, the median follow-up duration was limited to 4.3 years, constraining the evaluation of long-term outcomes. Furthermore, only a subset of patients had follow-up exceeding 20 years, potentially affecting the accuracy of late event and reintervention estimates. While these limitations are recognized, our findings underscore critical trends in the modern management of d-TGA. Consanguinity emerged as a significant risk factor, reinforcing the need for genetic counseling and community-level awareness, particularly in regions where consanguineous marriage remains prevalent. The arterial switch operation (ASO) continues to demonstrate superior long-term outcomes and should remain the cornerstone of d-TGA management. Importantly, the development of a national tertiary care center has substantially improved access to timely surgical intervention and continuity of care, eliminating the need for treatment abroad. As congenital cardiac surgery advances across the region, future studies exploring the interplay of genetic, environmental, and health system factors will be essential to further refine care and improve outcomes in this unique patient population.

Future work in the Gulf Cooperation Council (GCC) region should focus on establishing prospective registries and multicenter collaborations to validate genetic and environmental risk factors, particularly consanguinity and maternal diabetes. Standardized surveillance protocols are also needed for arterial switch operation survivors, with structured monitoring of valve function and coronary artery patency to guide timely intervention and ensure durable outcomes.

## Data Availability

The raw data supporting the conclusions of this article will be made available by the authors, without undue reservation.
